# The Central New York Medical Association

**Published:** 1888-12

**Authors:** 


The Central New York Medical Association held its regular semi-annual meeting in Rochester, November 20, 1888. The Association celebrated its twenty-first birthday in a fitting manner. Dr. E. M. Moore, its first President, delivered an interesting address, in which he reviewed his surgical work, embracing a period of upwards of forty years, during which he had been a teacher. This was the best attended meeting the Association ever held, and we hope to publish a full account of its proceedings in our January number.

